# Development of a plant-based oral vaccine candidate against the bovine respiratory pathogen *Mannheimia haemolytica*

**DOI:** 10.3389/fpls.2023.1251046

**Published:** 2023-09-15

**Authors:** Angelo Kaldis, Muhammed Salah Uddin, Jose Ortiz Guluarte, Coby Martin, Trevor W. Alexander, Rima Menassa

**Affiliations:** ^1^ London Research and Development Centre, Agriculture and Agri-Food Canada, London, ON, Canada; ^2^ Lethbridge Research and Development Centre, Agriculture and Agri-Food Canada, Lethbridge, AB, Canada; ^3^ Department of Agricultural, Food and Nutritional Science, University of Alberta, Edmonton, AB, Canada; ^4^ Department of Biology, Western University, London, ON, Canada

**Keywords:** bovine respiratory disease, plant-made vaccine, oral vaccine, Mannheimia haemolytica, molecular farming, mucosal immunity, agroinfiltration

## Abstract

Bovine respiratory disease (BRD) affects feedlot cattle across North America, resulting in economic losses due to animal treatment and reduced performance. In an effort to develop a vaccine candidate targeting a primary bacterial agent contributing to BRD, we produced a tripartite antigen consisting of segments of the virulence factor Leukotoxin A (LktA) and lipoprotein PlpE from *Mannheimia haemolytica*, fused to a cholera toxin mucosal adjuvant (CTB). This recombinant subunit vaccine candidate was expressed in the leaves of *Nicotiana benthamiana* plants, with accumulation tested in five subcellular compartments. The recombinant protein was found to accumulate highest in the endoplasmic reticulum, but targeting to the chloroplast was employed for scaling up production due the absence of post-translational modification while still producing feasible levels. Leaves were freeze dried, then orally administered to mice to determine its immunogenicity. Sera from mice immunized with leaf tissue expressing the recombinant antigen contained IgG antibodies, specifically recognizing both LktA and PlpE. These mice also had a mucosal immune response to the CTB+LktA+PlpE protein as measured by the presence of LktA- and PlpE-specific IgA antibodies in lung and fecal material. Moreover, the antigen remained stable at room temperature with limited deterioration for up to one year when stored as lyophilized plant material. This study demonstrated that a recombinant antigen expressed in plant tissue elicited both humoral and mucosal immune responses when fed to mice, and warrants evaluation in cattle.

## Introduction

Bovine respiratory disease (BRD) is the most common and costly disease in North American feedlot cattle (Blakebrough-Hall et al., 2022). A 2010 survey of the United States cattle industry found that 26.5% of all non-predator-related deaths, a total of 460 000 cattle, resulted from BRD (USDA, 2011). As a result, economic losses in North American feedlots have been approximated to be greater than $500 million per year (Miles, 2009). Although multifactorial in nature, BRD is mainly caused by opportunistic bacterial pathogens which colonize the upper respiratory tract and is more likely to occur shortly after feedlot placement (Snyder and Credille, 2020). Stressors occurring from feedlot placement, including transportation, comingling, and primary viral infections (Rice et al., 2007; Timsit et al., 2016) contribute to innate immunity suppression by altering calf mucosal secretions, anionic peptides and β-defensins (Brogden et al., 1998). This contributes to bacterial pathogen proliferation in the upper respiratory tract, and translocation and infection in the lungs (Timsit et al., 2020). Current BRD mitigation strategies include antimicrobial treatment upon feedlot arrival (metaphylaxis) and vaccination against BRD pathogens. However, antimicrobial resistance has increased in BRD-associated bacterial pathogens as a result of broad antimicrobial use, and vaccines have afforded variable protection (Lemon and Mcmenamy, 2021; Andres-Lasheras et al., 2022). Therefore, novel methods to mitigate BRD pathogens, including new vaccines, are needed.

While several bacterial pathogens are implicated in BRD, *Mannheimia haemolytica* is a principal one, often detected in acute pneumonia cases (Booker et al., 2008). *M. haemolytica* are non-motile, gram-negative, coccobacilli that primarily inhabit the nasopharynx and tonsils of ruminants (Frank et al., 1995). Of the 12 *M. haemolytica* serotypes, the majority of BRD cases are associated with serotype A1 affecting approximately 75% of feedlot cattle in North America (Snyder and Credille, 2020). Consequently, efforts to understand and mitigate this pathogen have been strongly directed towards this serotype. The pathogenicity of *M. haemolytica* is in part due to the virulence factor leukotoxin (Lkt), a member of the repeats-in-toxin (RTX) family of cytotoxins. Lkt has detrimental effects on macrophages and other leukocytes, and more severe effects on neutrophils (Snyder and Credille, 2020).

There are currently several commercial vaccines available against *M. haemolytica*. These vaccines come in the form of cell culture supernatants, bacterin-toxoid, and extracted antigens, while they are administered via intramuscular, subcutaneous and more recently intranasal routes (Rice et al., 2007; Confer and Ayalew, 2018). However, the results of these vaccines in controlled trials have been inconsistent and yielded limited efficacy in production settings (Rice et al., 2007; Larson and Step, 2012). The relative success of protein-based, cell-free vaccines led to the development of *M. haemolytica* protein subunit vaccines which are comprised of partial antigens, intended to elicit specific antibody responses. Potential targets for vaccine development include virulence factors such as Lkt, as well as surface lipoproteins such as PlpE. An intranasal vaccination of cattle with a PlpE-LKT-cholera toxin subunit B chimeric protein (SAC102) produced in *E. coli* and purified by immobilized metal affinity chromatography (IMAC) stimulated serum and nasal antibodies in vaccinated calves, and intrabronchial challenge in these calves produced fewer clinical symptoms than unvaccinated animals (Ayalew et al., 2009). Nevertheless, microbial-produced antigens for cattle require administration to individual animals and thus have high labor demands associated with their use.

Alternatively, plants have been explored in the past 30 years as bioreactors for the production of recombinant proteins. Plants are an excellent vehicle for the production of veterinary subunit vaccines targeted for oral mucosal delivery since farm animals consume plants as part of their diet and mass medication could be achieved through their use (Rybicki, 2018). There are several reports of oral vaccines produced in plants with demonstrated efficacy (Miletic et al., 2015; Shahid and Daniell, 2016; Sanchez-Lopez et al., 2021). It has been argued that the plant cell walls bioencapsulate antigens and protect them from degradation in the stomach (Kwon and Daniell, 2016; Daniell et al., 2019). In fact, it has been reported that plant tissue might prolong the residence time on the mucosa, thereby increasing antigen uptake and enhancing the immune response (Merlin et al., 2017; Sanchez-Lopez et al., 2021). Furthermore, oral delivery of subunit vaccines is less expensive than parenteral immunization as it obviates the need for purification which can constitute 70-80% of the cost of producing the vaccine (Tuse et al., 2014; Tschofen et al., 2016). This also reduces stress on the animals, reducing hands-on management, and promoting broad vaccination of farm animal populations (Kolotilin et al., 2014).

In this study, we fused segments of Lkt and PlpE to the B-subunit of the cholera toxin (CTB) to enhance mucosal immunity. This antigen was produced in the leaves of *Nicotiana benthamiana* plants, which were subsequently freeze-dried and orally administered to mice for evaluation of the immune response.

## Results and discussion

### Vaccine candidate design and production in *N. benthamiana*

**Figure 1:**
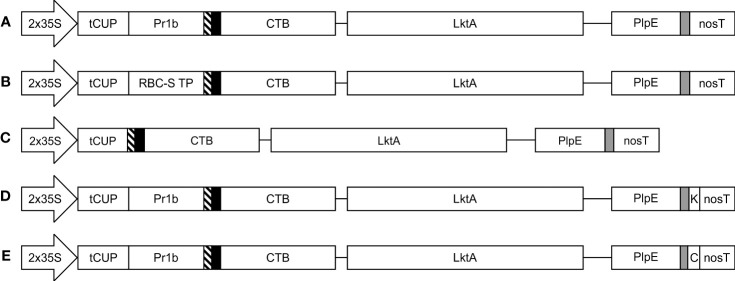
Schematic representation of the constructs used in the *Agrobacterium*-mediated transient expression of CTB+LktA+PlpE in *N. benthamiana* leaves. All five constructs are under the control of the double-enhanced cauliflower mosaic virus 35S promoter (2x35S), a translational enhancer from the tobacco cryptic upstream constitutive promoter (tCUP), and the nopaline synthase terminator (nosT). Recombinant protein accumulation was targeted to the **(A)** apoplast; **(B)** chloroplast; **(C)** cytoplasm; **(D)** endoplasmic reticulum; and **(E)** vacuole. Pr1b denotes the tobacco pathogenesis-related 1b signal peptide; RBC-S TP is the transit peptide from the small subunit of RuBisCo; thin blocks with diagonal lines, solid black or grey filling are Xpress, HA, and c-Myc tags, respectively. K represents the KDEL endoplasmic reticulum retrieval signal, while C is the barley lectin vacuole targeting signal. The CTB and LktA sequences are joined by a GPGP linker while the LktA and PlpE sequences are connected through a 3x(GGGS) linker. Construct segments are not drawn to scale.

The structural gene of the cytolytic leukotoxin operon, LktA, and the outer membrane *Pasteurella* lipoprotein E (PlpE) had been previously shown to be part of effective subunit vaccines against *M. haemolytica* when produced in *E. coli* (Ayalew et al., 2008; Ayalew et al., 2009; Confer et al., 2009a; Confer et al., 2009b). Using a similar recombinant protein approach, we created a fusion protein beginning with the B subunit of cholera toxin (CTB) from *Vibrio cholerae* at the N-terminus (**Figure 1**). CTB acts as a strong mucosal adjuvant and is thought to facilitate protein uptake into epithelial cells, resulting in its immune-recognition and response, leading to potentially improved efficacy of vaccines (Sheoran et al., 2002; Holmgren et al., 2005). Indeed, a similar CTB-LktA protein was shown to bind the cholera toxin receptor GM1-ganglioside, highlighting the binding activity of the CTB component (Ayalew et al., 2009). We used a larger region of the LktA gene in our construct than the previously reported sequence (Ayalew et al., 2008), and fused it to the C terminus of CTB with a GPGP amino acid linker to separate the 2 domains. The LktA domain consisted of the nucleotides encoding amino acids 705 through to the C-terminus, amino acid 953, of the *M. haemolytica* LktA gene (GenBank: M20730.1). This region was selected based on previous work that used amino acids 719-939, which encompassed the neutralizing and non-neutralizing antibody epitopes (Rajeev et al., 2001). We included more amino acids on both sides of this segment, which were highly conserved among multiple serotypes of *M. haemolytica*, with the intent of providing further stability to the resultant protein and greater antigen recognition across multiple serotypes. The LktA segment was followed by a linker (GGGS)_3_ and the immunodominant surface epitope (R2) of the PlpE gene. Antibodies against R2 were previously shown to sufficiently stimulate complement-mediated bacteriolysis (Ayalew et al., 2004).

The subcellular localization of a protein can affect its accumulation, and often proteins will not accumulate at all in some subcellular compartments (Pereira et al., 2014). For this reason, we explored targeting the CTB+LktA+PlpE fusion protein to five subcellular compartments: the apoplast (APO), chloroplast (CHL), cytoplasm (CYT), endoplasmic reticulum (ER), or vacuole (VAC) using expression vectors previously tested in our lab to direct the green fluorescent protein (GFP) to the correct subcellular compartment (Conley et al., 2009). The five expression constructs were transformed into *Agrobacterium tumefaciens* then co-infiltrated into *N. benthamiana* leaves with an *Agrobacterium* strain containing p19, a suppressor of post-transcriptional gene silencing from *Cymbidium* ringspot virus (Silhavy et al., 2002). To determine when the accumulation of the recombinant protein peaked after Agroinfiltration, a time course was conducted. Samples were collected from each of five infiltrated plants 4, 6, and 8 days post-infiltration (dpi) on three separate occasions (15 biological replicates). Each was extracted, then analyzed by SDS-PAGE and western blot to determine the accumulation levels of antigen in the subcellular targets. Data were expressed as averages of all the samples for each compartment.

**Figure 2:**
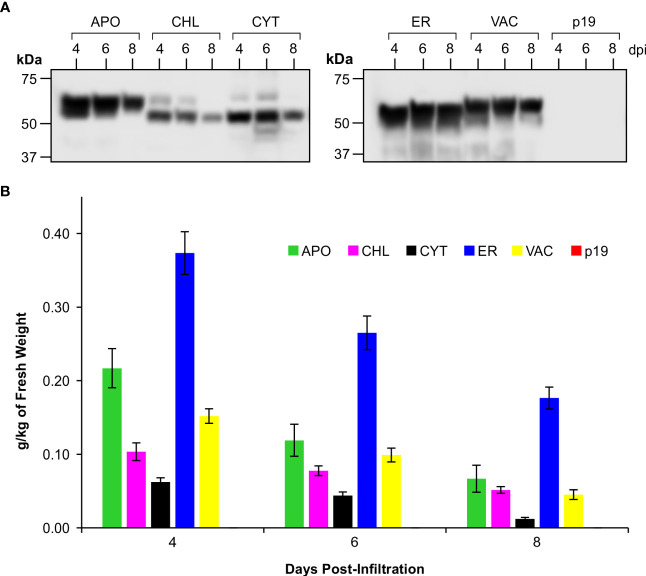
Accumulation of CTB+LktA+PlpE recombinant protein in various subcellular compartments following transient expression in the leaves of *N. benthamiana*. **(A)** Western blot hybridization of extracts pooled from 5 plants co-infiltrated with *A tumefaciens* containing constructs of p19 and of CTB+LktA+PlpE targeted to the apoplast (APO), chloroplast (CHL), cytoplasm (CYT), endoplasmic reticulum (ER), or vacuole (VAC). Blots were detected with an anti-c-Myc antibody which only recognizes a C-terminal tag on the CTB+LktA+PlpE protein. **(B)** Average recombinant protein accumulation levels in the five subcellular compartments as measured in 5 individual plants over three separate sets of infiltrations (n=15). Data are expressed as grams of recombinant protein per kilogram of fresh leaf weight at 4, 6, and 8 dpi. Error bars = ± standard error of the mean.

Western blots of pooled extracts (**Figure 2A**) demonstrated the size variability in the products that were detected, but also showed that recombinant protein successfully accumulated in all five of the tested subcellular compartments of *N. benthamiana* leaves. However, we observed that the CTB+LktA+PlpE fusion protein accumulated to its highest level in all compartments on day 4 post-infiltration, and that its levels dropped with time (**Figure 2B**). Depending on the protein’s stability, conformation, and post-translational modifications, some proteins will become more or less abundant over the first week or two before they disappear completely. Some proteins remain stable over 3-8 days (Chin-Fatt et al., 2021; VanderBurgt et al., 2023), some increase their accumulation with time (Saberianfar et al., 2019), and others reach their highest accumulation at 4-6 days and then go down with time (Garabagi et al., 2012). Here, we found that accumulation levels are highest four days post-infiltration, and this is the time we subsequently harvested the infiltrated leaf material.

The conventional threshold of economic viability for recombinant protein production in plants is considered to begin at 0.1 g/kg of fresh weight (FW) of plant material (Schiefer et al., 1978; Rybicki, 2009; Saberianfar et al., 2019; Schillberg et al., 2019; Sanchez-Lopez et al., 2021; Schillberg and Finnern, 2021). The CTB+LktA+PlpE fusion protein achieved this benchmark in 4 of the 5 targeted subcellular compartments. Accumulation in each compartment peaked at 4 dpi, with the highest levels measured at 0.37 g/kg FW in the endoplasmic reticulum (ER), 0.22 g/kg FW in the apoplast (APO), 0.1 g/kg FW in chloroplasts (CHL), and 0.15 g/kg FW in the vacuole (VAC) (**Figure 2B**). The yield in the cytoplasm (CYT) was below the conventional threshold with a level of 0.06 g/kg FW, and was not considered going forward.

### The plant-produced fusion protein is glycosylated in the secretory pathway

**Figure 3:**
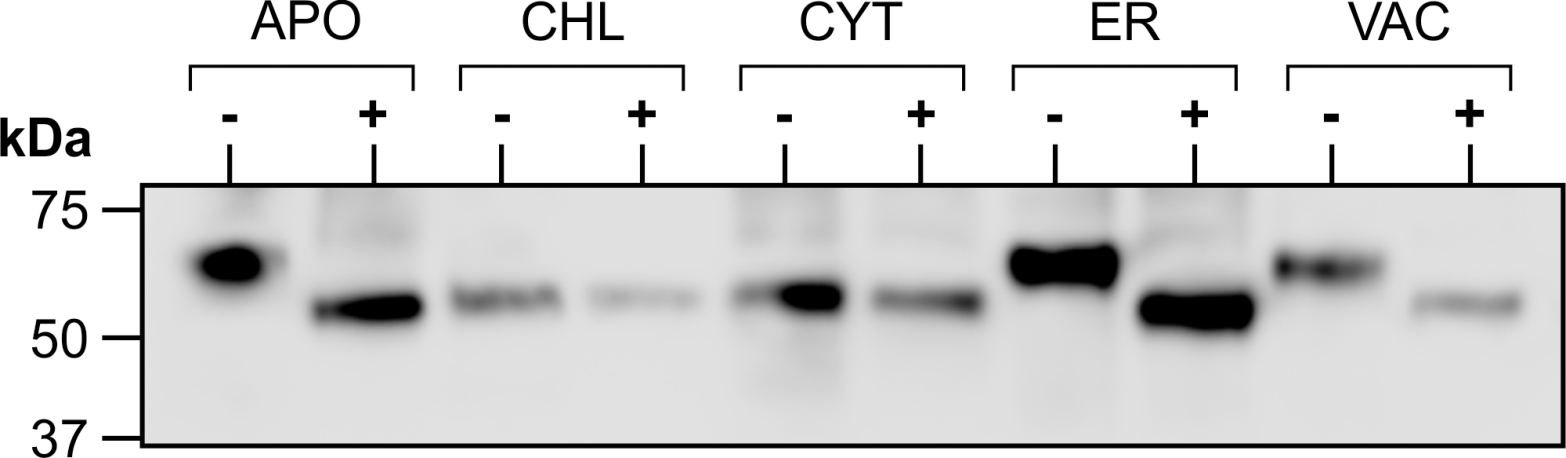
Glycosylation of transiently expressed CTB+LktA+PlpE. Extracts from *N. benthamiana* transiently expressing each of the five subcellularly-targeted constructs of CTB+LktA+PlpE were digested with PNGase F (+) or incubated without the enzyme (-), then analyzed by western blot.

Since the accumulation levels of our fusion protein reached higher amounts in the secretory pathway, we considered the potential impact of glycosylation on this synthetic protein comprised exclusively of prokaryotic sequences. The heterogeneity in size of the recombinant protein in **Figure 2A** indicated that glycosylation was likely occurring in the secretory pathway. The predicted molecular weight of the fusion protein based on its amino acid sequence is 49.2 kDa, corresponding to the band observed in the CHL- or CYT-targeted proteins. Indeed the cytosol or chloroplasts do not have the machinery to glycosylate proteins. To test whether the recombinant protein targeted to the secretory pathway (ER, APO or VAC) is glycosylated, crude extracts were digested with PNGase F, separated by SDS-PAGE and detected by western blot. No shift in size was detected with the CHL- or CYT-targeted protein. However, a decrease in size was seen in the product when accumulation was targeted to the APO, ER and VAC (**Figure 3**). This demonstrates *N*-glycosylation of the recombinant fusion protein in the secretory pathway. The observed mass of the cleaved products from the APO, ER, and VAC, and the non-glycosylated protein in the CHL and CYT was approximately 50 kDa, close to the predicted 49.2 kDa molecular weight of the fusion protein. It is possible that the higher accumulation of the protein in the secretory pathway may be due to the presence of glycans that could shield the protein from proteolysis. However, the presence of those glycans may also shield the antigens from the immune system.

### Scaling up production and stability of chloroplast-targeted antigen

**Figure 4:**
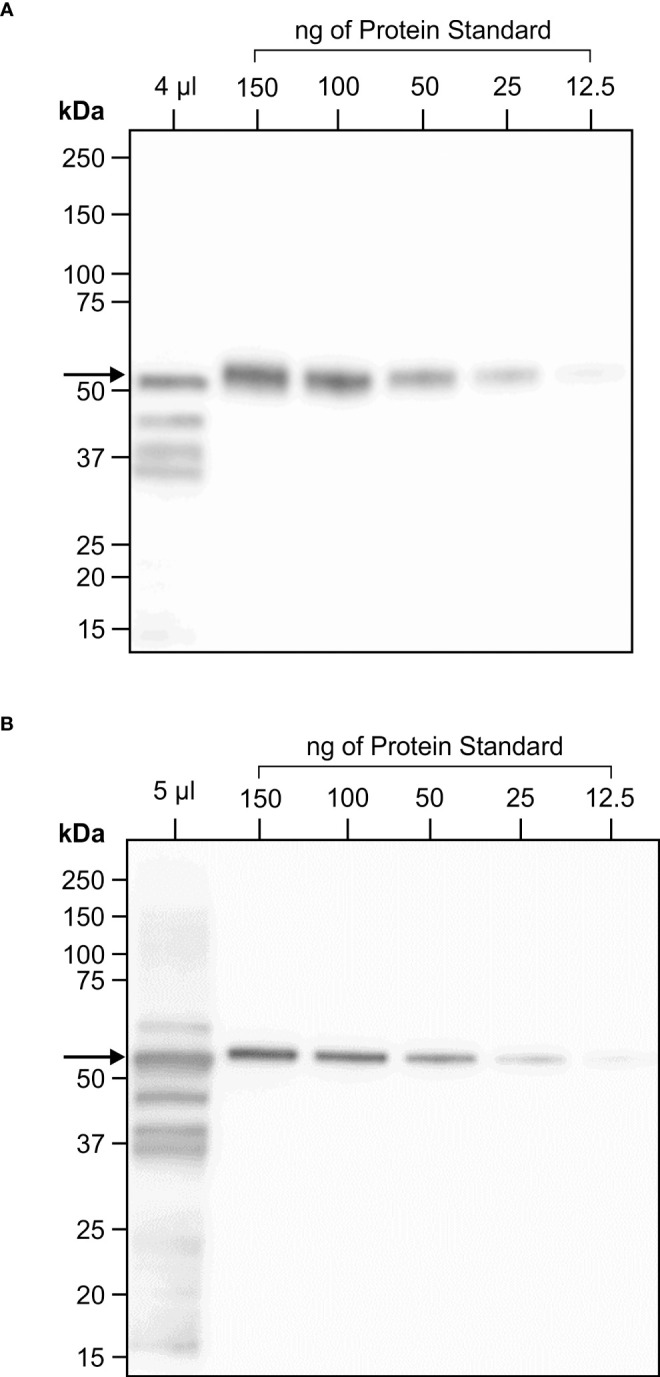
One year stability of transiently expressed CTB+LktA+PlpE recombinant protein in freeze dried *N. benthamiana* leaf tissue. **(A)** Western blot hybridization of extract from a pooled, freeze dried sample of *N. benthamiana* plants infiltrated with *A tumefaciens* containing constructs of p19 and of CTB+LktA+PlpE targeted to the chloroplast. The 4 µl loaded in the first lane represent the amount of soluble protein in 0.206 mg of lyophilized tissue. **(B)** Western blot hybridization of extract prepared from the same pool of infiltrated, freeze dried, *N. benthamiana* leaves that was milled following its storage in a sealed container at ambient temperature for one year. The 5 µl loaded in the first lane represent the amount of soluble protein in 0.254 mg of lyophilized tissue. Both blots were detected with an anti-HA antibody which only recognizes an N-terminal tag on the CTB+LktA+PlpE protein.

Considering the prokaryotic origin of the three CTB+LktA+PlpE segments and the lack of glycosylation in their native forms, we scaled up production of the fusion protein targeted to the chloroplast to avoid potential interference of glycans with protein folding or masking immunogenic epitopes. *Agrobacterium-*infiltrated *N. benthamiana* leaves were harvested 4 days post-infiltration, frozen and lyophilized. Western blot analysis was performed on protein extracted from the lyophilized tissue to determine the amount of recombinant protein in the batch of dried leaves. On the blots, we observed a full-size band at about 50 kDa, and three smaller bands which may have resulted from protein cleavage during lyophilization. The full-size band alone was quantified against known amounts of a standard protein (**Figure 4A**). There was 6.1 mg CTB+LktA+PlpE in 19.5 g of lyophilized *N. benthamiana* leaves.

The stability of the CTB+LktA+PlpE antigen within the cells of the freeze dried *N. benthamiana* leaves was assessed again immediately before its use in the immunization of mice. The lyophilized leaf tissue had been stored in a sealed container at ambient temperature for 12 months prior to pulverization using a ball-grinding mill. Soluble protein from a sample of this milled material was extracted and analyzed by SDS-PAGE and western blot hybridization in the same fashion that was employed when the tissue was originally infiltrated and freeze dried (**Figure 4B**). There was little change in the amount of full-length product present when compared to the original quantification one year earlier. This high antigen stability of a plant-based vaccine has also been reported by Lee et al. (2008). In that study, antigen containing the plant material was oven-dried for a week at 50° C and was reported to be stable at ambient temperature for up to one year (Lee et al., 2008). The lack of need for refrigeration adds practicality to the administration of this potential vaccine to cattle.

### Antigen-specific antibody production in mice

**Figure 5:**
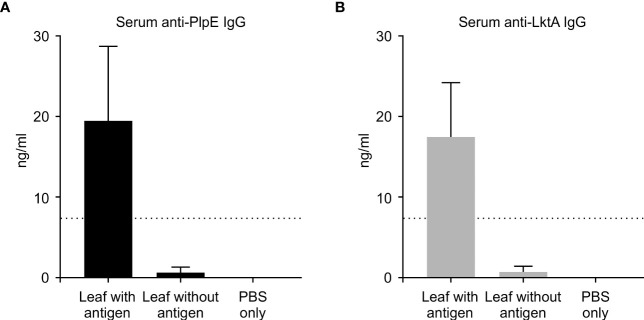
Systemic immune response in mice measured by ELISA on day 70. ELISA plates coated with either recombinant PlpE or LktA protein were used to measure **(A)** anti-PlpE and **(B)** anti-LktA antibodies in sera from immunized mice. Immune responses from three treatment groups were compared: 1) leaf with antigen (Intra-gastric inoculation of *Agrobacterium*-infiltrated, freeze dried, pulverized leaf tissue containing CTB+LktA+PlpE protein; mixed with PBS); 2) leaf without antigen (Intra-gastric inoculation of *Agrobacterium*-infiltrated, freeze dried, pulverized leaf tissue containing no protein; mixed with PBS); and 3) PBS only/control mice (Intra-gastric inoculation of PBS without any plant material or protein). The dashed line indicates the limit of detection (≥ 7.8 ng/ml). Results are expressed as mean ± standard error.

**Figure 6:**
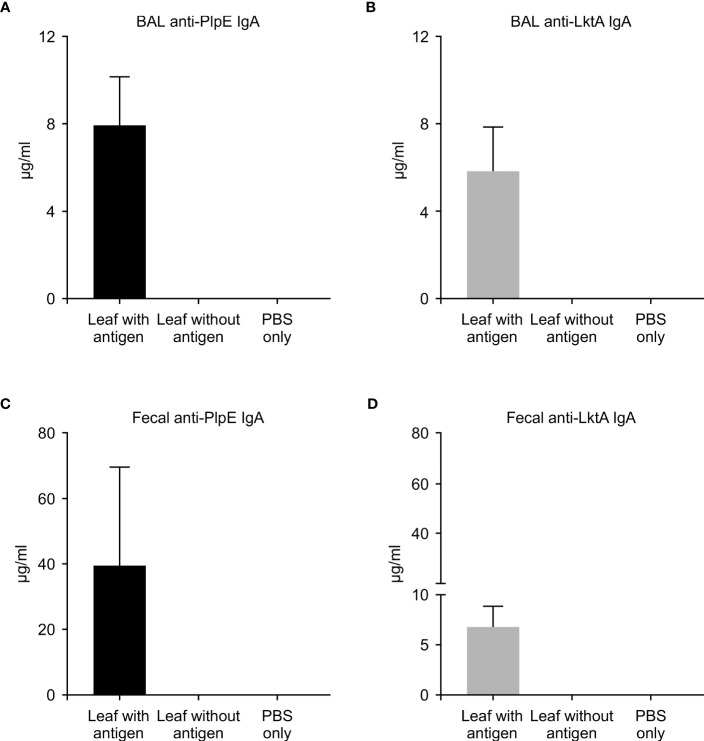
Mucosal immune response in mice measured by ELISA on day 70. ELISA plates coated with either recombinant PlpE or LktA protein were used to measure **(A)** anti-PlpE and **(B)** anti-LktA antibodies in bronchoalveolar lavage (BAL), and **(C)** anti-PlpE and **(D)** anti-LktA antibodies in feces from immunized mice. Fecal and BAL samples were collected and analyzed from three treatment groups of mice: 1) leaf with antigen (Intra-gastric inoculation of *Agrobacterium*-infiltrated, freeze dried, pulverized leaf tissue containing CTB+LktA+PlpE protein; mixed with PBS); 2) leaf without antigen (Intra-gastric inoculation of *Agrobacterium*-infiltrated, freeze dried, pulverized leaf tissue containing no protein; mixed with PBS); and 3) PBS only/control mice (Intra-gastric inoculation of PBS without any plant material or protein). Results are expressed as mean ± standard error.

To evaluate whether this plant expressed chimeric protein would elicit an immune response, mice were vaccinated by oral gavage of a slurry of leaf material containing 50 μg of the recombinant protein per dose (**Table 1**). Sera collected from mice 42 days post immunization did not have quantifiable levels of anti-PlpE or anti-LktA IgG (limit of quantification ≥ 7.8 ng/ml). However, on day 56, one animal from the leaf with antigen treatment group did exhibit an IgG response above the limit of quantification (data not shown). On day 70, all of the mice in the leaf with antigen treatment group displayed levels of both anti-PlpE and anti-LktA IgG (**Figure 5**). In contrast to this leaf with antigen treatment group, none of the mice administered only PBS had measurable IgG against either antigen. A single animal from the plant control leaf without antigen group (treatment 2) did show an antibody response on day 70, however it was below the limit of quantification, and likely due to background noise from the ELISA. Similar to IgG responses, anti-PlpE or anti-LktA IgA were only quantifiable in samples on day 70. An exception was the same animal in the leaf with antigen treatment group that had antigen-specific IgG on day 56, also had IgA against LktA and PlpE on day 56 in bronchoalveolar lavage (BAL) and fecal samples (data not shown). On day 70, mice from the leaf with antigen treatment had both anti-PlpE IgA and anti-LktA IgA in BAL and fecal samples, while the other groups did not have any detectable IgA antibodies against PlpE or LktA (P < 0.05; **Figure 6**). It is noteworthy that in our study, the antibody responses in BAL and feces were approximately a magnitude greater than sera IgG amounts. Combined, these data show that oral immunization of mice with lyophilized plant leaves expressing CTB+LktA+PlpE stimulated humoral and mucosal anti-PlpE and anti-LktA antibodies, indicating that the chimeric protein is immunogenic, and retained immunogenicity after oral administration. The combination of LktA and PlpE antigens were selected for use in our study because intranasal vaccination of calves with a similar chimera stimulated nasal antibodies after *M. haemolytica* challenge and enhanced protection against infection (Ayalew et al., 2009). In the study by Ayalew et al. (2009), the authors noted an increase in nasal anti-Lkt antibody responses, which was only significant after bacterial challenge, and not after the booster immunization alone. The next step will be determining whether the plant CTB+LktA+PlpE is similarly immunogenic in calves after in-feed administration.

**Table 1:** Experimental design of mouse trial. Balb/c pathogen-free mice (n=18 mice per treatment) were immunized at 14-day intervals by oral gavage.

	Day 0	Day 14	Day 28	Day 42	Day 56	Day 70
Immunization timepoints	**✔**	**✔**	**✔**	**✔**	**✔**	**-**
Number of mice sacrificed	**-**	**-**	**-**	6	6	6
Samples collected	**-**	**-**	**-**	Blood, BAL, Feces	Blood, BAL, Feces	Blood, BAL, Feces

Six mice per treatment were sacrificed on days 42, 56 and 70 for collection of blood, feces, and bronchoalveolar lavage (BAL) samples.

While commercial vaccines exist for *M. haemolytica*, their subcutaneous or intranasal administration requires direct animal handling. An efficacious orally administered plant vaccine would be advantageous by allowing mass vaccination with reduced labor input, and lower production costs due to elimination of expenses associated with purification and formulation of the vaccine. Purification costs account for 70-80% of the cost of production of pharmaceutical proteins (Tuse et al., 2014; Tschofen et al., 2016). To date however, there are few reports of oral vaccines targeting BRD pathogens. Lee et al. (2008) evaluated transgenic alfalfa expressing the *M. haemolytica* GS60 outer membrane protein in rabbits. The authors reported that two oral immunizations with dried transgenic alfalfa resulted in seroconversion in one of six animals vaccinated. The low seroconversion may have been due to only two vaccinations being administered. Indeed, only one mouse showed seroconversion after four oral vaccinations in our study, and the number increased to all mice showing seroconversion after five immunizations. It is also possible that the varying immune responses reported in these studies stem from dissimilarities in the antigen design, the schedule of immunization, the adjuvant used, and the animal species being utilized (mice versus rabbits). Thus, it is important to determine the optimal number of immunizations for plant-based oral vaccines, especially concerning that differences in antigen structure and plant composition could affect digestion and antigen survival in the gastrointestinal tract.

In this study, we showed that when lyophilized leaf tissue expressing a putative subunit vaccine was orally administered to mice, the recombinant protein resisted degradation enough such that both LktA and PlpE antigenic components were taken up by M cells, allowing activation of immunocompetent cells in the mucosa-associated lymphoid tissue. Taghavian et al. (2013) have also found that Pichia-produced VP2 from inflammatory bursal disease virus survived digestion and induced an IgY immune response in chickens. The presence of antigen-specific secretory antibodies in lungs of mice indicated trafficking of stimulated immune cells from the gut occurred, while antigen-specific IgA in feces also showed a local response. Similarly, Shewen et al. (2009) found that feeding of transgenic alfalfa expressing Lkt50 led to an increase in Lkt-specific IgA in nasal secretions, though it could not be determined if immunity was stimulated in the lower gastrointestinal tract, or the upper respiratory tract through rumination and oropharyngeal exposure to antigen. Regardless, a plant-based vaccine could be targeted to confer protection against respiratory and gastrointestinal bovine pathogens. A vaccine delivering both respiratory and gastrointestinal protection against pathogens in calves would be highly valuable. Moreover, the antigen used in this plant-based vaccine remained stable for approximately one year while stored at room temperature. Thus the vaccine was shown to maintain long-term stability and elicit systemic and mucosal immunity in mice, and may have potential use as an in-feed vaccine in cattle to mitigate the BRD pathogen *M. haemolytica*.

## Materials and methods

### Construction and cloning of CTB+LktA+PlpE gene fusion

The sequence of CTB used is from the *Vibrio cholerae* toxin B subunit gene (GenBank: AY804244.1). The signal peptide region spanning the first 63 nucleotides was not included. Immediately following the CTB sequence, 12 nucleotides encoding a GPGP linker connected to the LktA segment. The LktA fragment consisted of the nucleotides encoding amino acids 705-953 of the *M. haemolytica* LktA gene (GenBank: M20730.1). The segment used in this CTB+LktA+PlpE construct was taken from a previously reported sequence (Rajeev et al., 2001) and expanded to include 14 amino acids on either side. This was followed by twelve amino acids, three repeats of GGGS, joining the 3’ end of the LktA segment to the nucleotides encoding the immunodominant 55-amino acid R2 region of PlpE from *M. haemolytica* sequence (Ayalew et al., 2008). This gene fusion was capped by a hemagglutinin tag at the 5’ end and a c-Myc tag at the 3’ end.

The gene was commercially synthesized (Bio Basic Inc., CAN) following optimization of codon usage throughout the sequence for nuclear expression in *N. benthamiana*. Flanking sequences necessary for Golden Gate cloning with BsaI were added to both ends of the gene. It was subsequently cloned into five modified versions of the pCaMterX plant expression vector (Menassa et al., 2001), which had been altered to accommodate Golden Gate cloning, pCLGG. Each version of pCLGG targeted recombinant protein accumulation to a different subcellular compartment: the apoplast (APO), chloroplast (CHL), cytosol (CYT), endoplasmic reticulum (ER), and vacuole (VAC) (**Figure 1**). Cloning into these expression vectors was done using NEB Golden Gate Assembly Mix (New England Biolabs Inc., USA) according to the manufacturer’s instructions. Cloning of the CTB+LktA+PlpE gene fusion into the five expression vectors was verified by DNA sequencing.

### Transient expression in *N. benthamiana* and western blot analysis

The five vectors targeting the CTB+LktA+PlpE gene fusion to various subcellular compartments were transformed into *Agrobacterium tumefaciens* (EHA105). *N. benthamiana* plants were grown and infiltrated as previously described (Miletic et al., 2015; Saberianfar et al., 2019). For these experiments, four leaves of five plants were infiltrated on three separate occasions. From each infiltrated plant, a leaf disc (7 mm diameter) was taken from each of the four leaves and pooled as one sample. Samples were collected from the infiltrated plants 4, 6, and 8 days post-infiltration (dpi). Total soluble protein (TSP) was extracted from each sample in 200 μl of plant protein extraction buffer (PEB) [1xphosphate-buffered saline (PBS), 0.1% (v/v) Tween-20, 2% (w/v) polyvinylpolypyrrolidone (PVPP), 100mM ascorbic acid, 1mM ethylenediaminetetraacetic acid (EDTA), 1mM phenylmethanesulfonylfluoride (PMSF),1 μg/ml leupeptin] as detailed in (Pereira et al., 2014). To aid in cell lysis, crude extracts were also sonicated on ice for 30 seconds at a 30% amplitude. Following centrifugation at 20,000 x *g* for 10 minutes at 4°C to clarify the lysates, TSP was quantified.

Samples of extracts from each infiltration were prepared for SDS-PAGE by mixing with a reducing, denaturing loading buffer before heat-denaturation at 100°C for 10 minutes before separation on 4-20% acrylamide, continuous gradient gels (Genscript, Piscataway, USA). Gels were subsequently transferred to polyvinylidene difluoride (PVDF) membranes using the TransBlot Turbo system (Bio-Rad, Hercules, USA). Recombinant protein levels in extracts were determined by comparison to a known protein standard on western blots probed with an anti-c-Myc antibody (Genscript, USA). The protein standard used was a recombinant fusion of eGFP with multiple tags including HA, c-Myc, and 6xHis, expressed in *N. benthamiana* and purified by immobilized metal affinity chromatography. Accumulation levels of the recombinant antigen in each subcellular compartment were averaged for all individual plants across all three repetitions (**Figure 2B**).

### Deglycosylation analysis

Enzymatic deglycosylation of transiently produced CTB+LktA+PlpE targeted to the five subcellular compartments was done by digestion of the various extracts using PNGase F (New England Biolabs, USA) according to the manufacturer’s instructions. Digestion was allowed to proceed at 37°C overnight before analysis of the products via SDS-PAGE and western blot with the anti-c-Myc antibody.

### Increased scale of infiltration for mass production and stability determination of putative vaccine

To scale up the production of chloroplast-targeted CTB+LktA+PlpE, 6- to 7-week old *N. benthamiana* plants were infiltrated with a mixture of *Agrobacteria* containing the expression construct and a construct to express p19, prepared as described for the five targeting constructs, using vacuum. As a negative control, plants were infiltrated with *Agrobacteria* containing only the p19 expression construct. Whole plants were inverted into the *Agrobacteria* suspension, completely submerging the leaves. A vacuum of 85 kPa was applied for 1 min. The vacuum was slowly released over 30 seconds, then plants were removed from the *Agrobacteria* mixture. Any leaves that were not infiltrated were removed. All infiltrated leaf material was collected and pooled 4 days post infiltration, flash frozen in liquid nitrogen, then freeze dried, which reduced the weight of the leaf material by approximately 90%. The dried leaf material was pulverized into small particles using a ball-grinding mill, then analyzed for recombinant protein integrity and amount per dry weight as determined by SDS-PAGE and western blot. The dried *N. benthamiana* leaves were stored for 12 months, and the stability of the recombinant protein was reassessed prior to the mice study by SDS-PAGE and Western blotting.

### Oral administration of candidate vaccine to mice

The immunogenicity of recombinant CTB+LktA+PlpE protein was studied using pathogen-free Balb/c (Charles River) mice as the experimental animal model. Mice were housed at the vivarium of Lethbridge Research and Development Centre (LeRDC) and work described in this study was conducted in accordance with the Canadian Council on Animal Care guidelines (https://ccac.ca/Documents/Standards/Guidelines/Farm_Animals.pdf) under protocol # 1829 approved by the animal care committee of LeRDC. A total of 54 mice were divided into three experimental groups (n=18 mice per treatment): 1) Leaf with antigen: *Agrobacterium*-infiltrated, dried leaf material expressing both p19 and CTB+LktA+PlpE (50 μg recombinant protein/160 mg total dry weight/mouse, mixed in 0.5 ml PBS), 2) Leaf without antigen: *Agrobacterium*-infiltrated, dried leaf material expressing only p19 (160 mg total dry weight, mixed in 0.5 ml PBS), and 3) PBS only: PBS without any leaf material or any antigen (0.5 ml PBS). All treatments were delivered by oral gavage, twice a day, with half of the treatment dose being administered in the morning, and the second half prepared and administered 6 h later. The Leaf with antigen-treated group received 50 µg of CTB+LktA+PlpE protein per immunization day. Mice were vaccinated by oral gavage on days 0, 14, 28, 42 and 56. Samples (blood, bronchoalveolar lavage, and fecal pellets) were collected on days 42, 56 and 70. At each sampling point, 6 mice from each group were sacrificed by exsanguination under deep anesthesia. To determine immune response, each sample was analyzed quantitatively for antigen-specific IgG and IgA.

### Quantification of antigen-specific serum IgG

Antigen-specific antibody (IgG) responses in serum were quantified by enzyme-linked immunosorbent assay (ELISA) as described (Uddin et al., 2023). Sequences corresponding to individual constructs (*i.e*. LktA and PlpE) were commercially synthesized (BioBasic Inc., Markham, Canada), codon optimized for expression in *Escherichia coli*, and subsequently purified by immobilized metal affinity chromatography. The purified recombinant antigens LktA and PlpE were used as ligands to coat ELISA plates at concentrations of 50 ng/well and incubated at 4°C overnight. After blocking with 50 mM Tris, 0.14 M NaCl, 1% BSA, pH 8.0 for 1 h at 37°C, serum samples (100 µL) were dispensed into wells of microtiter plates in duplicates, and incubated for 1 h. After washing and adding HRP-conjugated anti-mouse secondary IgG (Cat. No. A90-131P; Bethyl Laboratories Inc.), plates were incubated for 1 h at room temperature. Color development was performed by addition of the TMB substrate (3,3´,5,5´-tetramentylbenzidine; ThermoFisher). Reactions were stopped with 0.2 M H_2_SO_4_, and plates were read at 450 nm on a Microtiter Plate Reader (Synergy HTX Microplate Reader, BioTek). Standard curves were generated using a Mouse IgG ELISA Quantitation kit (Cat. No. E90-131; Bethyl Laboratories Inc.) where Mouse Reference Serum (RS10-101-6; Bethyl Laboratories Inc.) was used as standard.

### Quantification of secretory IgA (sIgA)

Bronchoalveolar lavage (BAL), and fecal samples were processed for IgA quantification after pre-processing of the samples as described (Hoang et al., 2008). Briefly, BAL samples were washed with PBS containing 0.1% (w/v) BSA and 1 mM of PMSF, followed by centrifugation. Freshly voided feces were collected and stored frozen at -80°C until use. Approximately 100 mg of feces was incubated in 400 µL PBS containing 0.05% Tween 20, 1% BSA, 1 mM PMSF and 0.1% Triton X-100 with vortexing to disrupt solid material. Samples were centrifuged at 17,000 x g for 10 min and supernatants were used for analysis. Following pre-processing, ELISA was used to measure the concentrations of mucosal secretory IgA (sIgA) in accordance with the Mouse IgA ELISA Quantitation kit (Cat. No. E90-103; Bethyl Laboratories Inc.). Briefly, 96-well ELISA plates were coated with 50 ng/well of purified recombinant LktA or PlpE and incubated at 4°C overnight. After blocking in 50 mM Tris, 0.14 M NaCl, 1% BSA, pH 8.0 for 1 h at room temperature, samples were applied in duplicate, then plates were incubated for 1 h at room temperature. After washing and the addition of HRP-conjugated anti-Mouse IgA Detection Antibody (A90-103P; Bethyl Laboratories Inc.), plates were further incubated for 1 h at room temperature. TMB substrate was added for color development, and finally reactions were stopped by adding ELISA stop solution. The plates were read at 450 nm to determine optical density on a spectrophotometric plate reader.

### Statistical analysis

Kruskal-Wallis test was performed to analyze the ELISA results. Significance was tested against the control by Kruskal-Wallis test with *post-hoc* Dunn’s multiple comparison test where p-value less than 0.05 was considered statistically significant. All analyses were performed using Microsoft Excel 2010 and GraphPad Prism version 9.4.1.

## Data availability statement

The original contributions presented in the study are included in the article/supplementary material. Further inquiries can be directed to the corresponding authors.

## Ethics statement

The animal study was approved by animal care committee of LeRDC. The study was conducted in accordance with the local legislation and institutional requirements.

## Author contributions

AK designed the constructs, carried out the plant transformation, and expression experiments. MU conducted the animal experiments and analysis. RM and TA conceptualized the study. JO participated in developing the animal use protocol and in mouse sampling. CM participated in construct design. AK, MU, RM, and TA co-wrote the manuscript. All authors contributed to the article and approved the submitted version.
